# Comparison of the Effects of Self-Administered Moxibustion versus Acupressure on Blood Pressure, Stress, Sleep Quality, and Quality of Life in Hypertensive Patients: A Randomized Parallel Trial

**DOI:** 10.3390/healthcare11152182

**Published:** 2023-08-01

**Authors:** Jongsin Jung, Jaehee Kim

**Affiliations:** Department of Alternative Medicine, Graduate School of Alternative Medicine, Kyonggi University (Seoul Campus), 24, Kyonggidae-ro 9-gil, Seodaemun-gu, Seoul 03746, Republic of Korea; jjs@kyonggi.ac.kr

**Keywords:** acupressure, hypertension, moxibustion, quality of life, self-care, sleep quality, stress

## Abstract

This study aimed to evaluate the effects of an 8-week self-administered moxibustion program on blood pressure, stress, sleep quality, and quality of life in hypertensive patients. We compared its effects with those of self-acupressure to assess the feasibility of using moxibustion as a self-management method for hypertension. Forty-six subjects were recruited and randomly assigned to a moxibustion group and an acupressure group. The moxibustion group applied moxa sticks to seven acupoints by themselves five times a week for 8 weeks, while the acupressure group placed acupressure patches on the same acupoints by themselves. Systolic and diastolic blood pressures were measured. Stress was assessed with plasma epinephrine, plasma norepinephrine, and the Perceived Stress Scale (PSS). Sleep quality and quality of life were evaluated using the Pittsburgh Sleep Quality Index (PSQI) and the World Health Organization Quality of Life-Brief Version (WHOQOL-BREF), respectively. Systolic and diastolic blood pressures decreased in the moxibustion and acupressure groups to a similar extent after the 8-week intervention. Plasma epinephrine and norepinephrine levels and PSQI variables were not changed in both groups. PSS decreased only in the moxibustion group. Quality of life related to physical health and environmental health, as assessed by the WHOQOL-BREF, significantly improved to a similar degree in both groups. Both self-administered moxibustion and acupressure therapies were effective in reducing blood pressure in hypertensive patients.

## 1. Introduction

Hypertension, defined as persistently high blood pressure, is the major cause of heart disease, stroke, and all-cause death globally [1,2]. Most hypertensive patients have primary hypertension, which has a multifactorial etiology including genetic predisposition and environmental and lifestyle risk factors [1]. Conventional treatments include first-line antihypertensive medications and adopting proper lifestyles, such as following an appropriate diet, engaging in regular exercise, ensuring adequate sleep, managing stress, being a non-smoker, and maintaining a healthy weight [1,2,3,4].

Although antihypertensive medications are effective in lowering blood pressure, some patients fail to adhere to medication and have uncontrolled blood pressure with medication, in some cases due to resistant hypertension, which poorly responds to medication [2,5]. Due to these reasons, along with the complex etiology of hypertension, multidisciplinary approaches, including various non-pharmacological options, have been recommended and applied to treat hypertension and manage its risk factors [5,6].

Hypertension is a common chronic disease that requires continuous treatment and management [7]. Self-care practices at home are known to have potential benefits for managing hypertension [8]. Therefore, a self-management method for hypertension with proven effectiveness can be helpful in the long-term management of the condition.

Among non-pharmacological treatments, acupoint stimulation therapies such as acupuncture, moxibustion, and acupressure have shown effectiveness in reducing blood pressure [9,10,11,12]. Unlike acupuncture, moxibustion and acupressure can be performed by the patients themselves [9,13]. Moxibustion involves using burning moxa to generate thermal stimulation, while acupressure utilizes finger pressure or a device [14,15]. A recent systematic meta-analysis study found that moxibustion therapy reduces blood pressure in hypertensive patients [12]. Additionally, a recent network meta-analysis study suggested that moxibustion may be more effective than acupuncture in lowering diastolic blood pressure in hypertensive patients [11]. Moxibustion could potentially have other effects in treating hypertension beyond acupoint stimulation [13,16,17]. It has been suggested that heat and fume generated during moxibustion may hypothetically affect vasodilatation and cardiac rhythm and result in mental relaxation, although the exact antihypertensive mechanism of moxibustion is not fully understood [13,16,17].

Taken together, moxibustion has demonstrated its effectiveness in lowering blood pressure [12,16]. Moreover, moxibustion is suggested as a promising self-treatment option for managing hypertension [12]. However, the effect of self-administered moxibustion on hypertension has not been investigated. Therefore, we conducted a study to explore the feasibility of using moxibustion as a self-management method for hypertension compared with self-acupressure, which is considered relatively noninvasive and safe [15].

In addition to managing blood pressure, it is important to consider quality of life in hypertensive patients, as they often experience a low quality of life compared to individuals with normal blood pressure [18]. However, the effect of moxibustion on the quality of life of hypertensive patients remains poorly understood.

Stress and poor sleep quality are well-known risk factors for hypertension and can contribute to a lower quality of life in hypertensive patients [3,19,20]. Hypertensive individuals often exhibit higher sympathetic nerve activity, which is associated with increased stress and poor sleep quality compared to normotensive individuals [21]. It is reasonable to assume that certain interventions may impact blood pressure and quality of life by reducing stress and sleep disturbances in hypertensive patients. However, only one study has investigated the effects of moxibustion on stress, sleep, and quality of life in hypertensive patients [16]. The authors reported no significant effects of the 4-week moxibustion therapy on sympathetic nerve activity, sleep quality, or quality of life [16].

Psychological stress is known to increase sympathetic nerve activity and stress hormone levels (i.e., cortisol, epinephrine, and norepinephrine), which can lead to elevated blood pressure [22]. However, there is limited evidence regarding the effectiveness of moxibustion on stress. Studies have reported the effects of moxibustion on stress hormones (i.e., cortisol) in stressed animal models with conditions like starvation and gastric ulcer [23,24], but its effects on stress hormones in human individuals are not yet established. The effect of moxibustion on mental stress is currently unknown. Furthermore, while improved sleep quality has been observed in patients with insomnia following moxibustion intervention [25], such effects have not been studied in hypertensive patients.

Accordingly, the purpose of this study was to verify the effectiveness of an 8-week self-administered moxibustion intervention in decreasing blood pressure, improving sleep quality and quality of life, and reducing perceived stress and epinephrine and norepinephrine levels in hypertensive patients. Additionally, we aimed to investigate whether self-moxibustion has greater effects on these outcomes compared to self-acupressure.

## 2. Materials and Methods

### 2.1. Study Design

This study was conducted as a parallel randomized clinical trial from January to June 2022. This study was approved by the Institutional Review Board of Kyonggi University (KGU-20210527-HR-070-02). Prior to participating in this study, all participants provided written informed consent. This study was registered on CRIS.nih.go.kr under the Korean Clinical Trial Registry (KCT0007424). Furthermore, this study adhered to the guidelines of the Consolidated Standards of Reporting Trials (CONSORT) [26].

**Figure 1:**
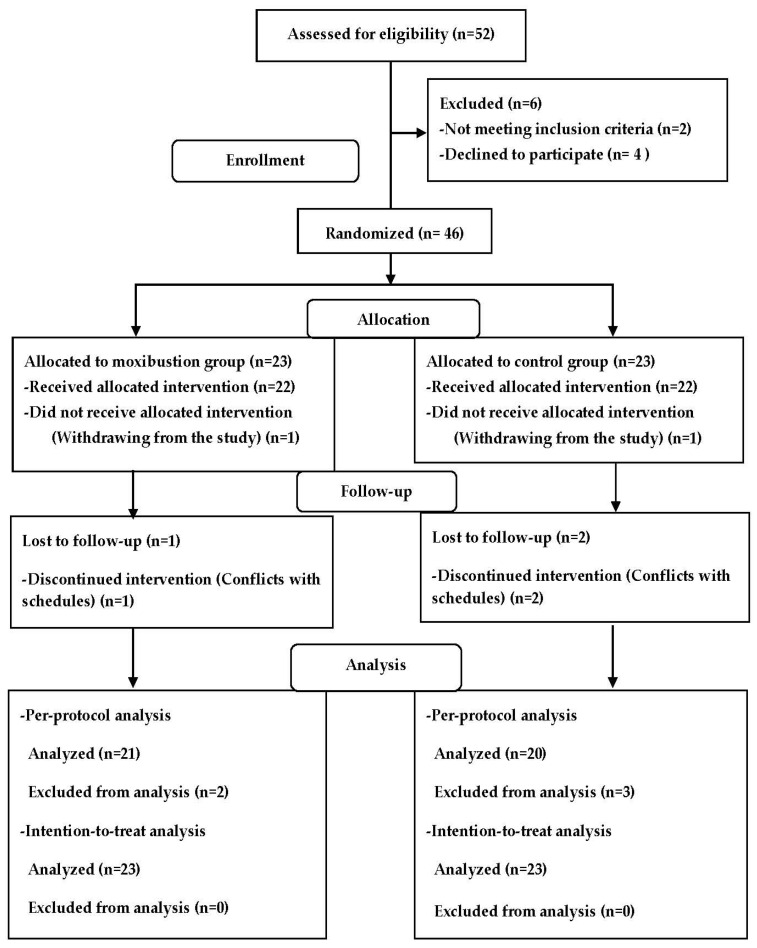
The study flowchart.

Subjects were recruited from the primary care clinic and continuing education center for moxibustion and meridian studies in Seoul, South Korea. A total of 52 participants were initially screened for eligibility based on inclusion and exclusion criteria. The CONSORT flowchart of this study is presented in Figure 1. Forty-six adult men and women were recruited and randomly assigned to the moxibustion group (*n* = 23) or the acupressure group (*n* = 23). Each participant selected a sealed and opaque envelope from a box, and their group assignment was determined by the labeled paper inside the envelope.

After the group assignment, two subjects withdrew from this study due to a change of mind. Therefore, a total of 22 subjects in each group underwent the intervention. Three subjects dropped out due to conflicts with their schedules. Finally, 21 subjects in the moxibustion group and 20 subjects in the acupressure group completed this study.

Each subject in the respective assigned group mastered the acupressure or moxibustion technique and underwent a consistency check to ensure proficiency. Adherence to antihypertensive medication and daily self-administration logs were recorded throughout the study. Systolic and diastolic blood pressures, perceived stress, sleep quality, and quality of life were measured at baseline and after 8 weeks. Additionally, blood samples were collected at baseline and after the 8-week intervention to evaluate levels of epinephrine and norepinephrine and a complete blood count.

### 2.2. Subjects

The inclusion criteria for this study were as follows: men and women aged 19 years or older; diagnosed primary hypertension (systolic blood pressure ≥ 140 mmHg or diastolic blood pressure ≥ 90 mmHg); currently taking antihypertensive medication; or having prehypertension (systolic blood pressure between 130 and 139 mmHg or diastolic blood pressure between 85 and 89 mmHg) [6]. The exclusion criteria included pregnancy, infectious disease, cancer, neurological disease, current use of psychological medications, or undergoing any acupoint stimulation therapy within the last 6 months.

### 2.3. Interventions

**Figure 2:**
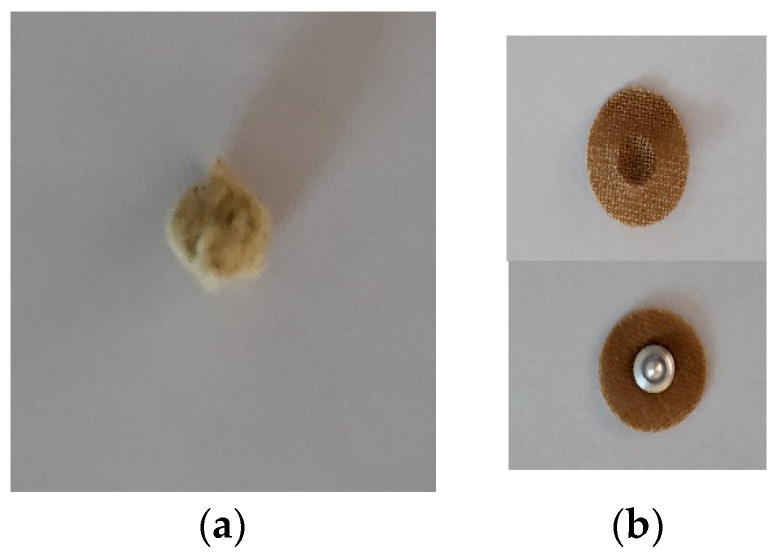
Moxibustion and acupressure devices: (**a**) Moxa stick; (**b**) Acupressure patch.

Subjects in the moxibustion group self-administered the treatment by applying 5 moxa cones (approximately half the size of a rice grain) (Figure 2a) directly on 7 acupoints located on the abdomen, arms, and legs. This self-administration was performed once a day, five days a week, for 8 weeks at their own homes. Each treatment session lasted approximately 15 min. The acupoints included CV4 (Gate of Origin in English; Guan Yuan in Chinese), CV6 (Sea of Qi; Qi Hai), and CV12 (Middle Cavity; Zhong Wan) located in the abdomen; LI11 (Pool at the Crook; Quchi); and ST36 (Leg Three Miles; Zusanli) located on both sides of the arm and leg. These acupoints have been used for hypertension in previous studies [12,13,16].

Subjects in the acupressure group self-applied acupressure patches, which had a metal stud pressing down on the acupoints (Figure 2b), on the same 7 acupoints for 15 min once a day, five days a week, for 8 weeks.

### 2.4. Measurements

The measurements included blood pressure, levels of epinephrine and norepinephrine, a complete blood count, perceived stress levels, sleep quality, quality of life, and adverse events. The resting systolic and diastolic blood pressures were measured in the morning using the auscultation method with a mercury sphygmomanometer and a stethoscope (Hico Medical Equipment Co., Ltd., Kyoto, Japan). Before the measurement, each subject was comfortably seated and allowed to relax quietly for 10 min. Blood pressure was then measured with the subject in a seated position, with their arm resting on a table and positioned at the level of the heart. The cuff was placed on the bare upper arm and adjusted according to the individual’s arm circumference.

The blood samples were sent to a commercial laboratory (Samkwang Medical Laboratories, Seoul, Republic of Korea) for a complete blood count and catecholamine analysis. Plasma samples were prepared by centrifuging the blood samples at 3000 rpm for 10 min, and they were collected using anticoagulant (EDTA)-treated tubes. The plasma epinephrine (pg/mL) and norepinephrine (pg/mL) levels were measured using high-performance liquid chromatography with electrochemical detection. This analysis was conducted using plasma catecholamine reagent kits (Chromsystems Instruments & Chemicals GmbH, Munich, Germany) and an electron capture detector (1260 LC–ECD, Agilent Technologies Inc., Santa Clara, CA, USA).

The subjective stress level was evaluated using the Korean version of the Perceived Stress Scale (PSS), which consists of 10 items [27]. Each item is scored on a scale of 0 to 4, and the total score ranges from 0 to 40, with higher scores indicating higher levels of stress [28]. The validity and reliability of the Korean PSS have been well established [29]. In this study, the reliability coefficient (Cronbach’s alpha) for the PSS was 0.68 at baseline and 0.72 after the intervention.

Sleep quality was assessed using the Pittsburgh Sleep Quality Index (PSQI), which measures subjective sleep quality over the past month [30]. The total score on the PSQI ranges from 0 to 21, with higher scores indicating poorer sleep quality [30]. The PQSI consists of 7 subscales that evaluate subjective sleep quality, sleep latency, sleep duration, habitual sleep efficiency, sleep disturbance, use of sleeping medication, and daytime dysfunction [31]. Each subscale is scored on a scale of 0 to 3 [30]. The Korean version of the PSQI has been found to be both valid and reliable [31]. In this study, Cronbach’s alpha for the PSQI total score was 0.64 at baseline and 0.54 after the intervention.

Quality of life was evaluated using the Korean brief version of the World Health Organization Quality of Life (WHOQOL-BREF) questionnaire [32]. The WHOQOL-BREF consists of 4 domains: physical health, psychological health, social relationships, and environment [33]. Higher scores indicate a better quality of life [33]. The domains consist of varying numbers of items, and the scores were transformed to a scale ranging from 0 to 100 for comparison across domains [33]. The Korean version of the WHOQOL-BREF has demonstrated good validity and reliability [34]. The Cronbach’s alpha values for the WHOQOL-BREF domains before and after the intervention ranged from 0.61 to 0.86.

Adverse events related to the intervention were recorded. Furthermore, the complete blood count analysis included measurements of red blood cells, white blood cells, platelets, hemoglobin, and hematocrit (%).

### 2.5. Sample Size

We planned to utilize a two-way analysis of variance (ANOVA) with repeated measures to examine the significance of intergroup differences in changes from baseline. Sample size calculation was performed using the G*Power program, version 3.1.9.4, to detect the significant interaction of the 2-way ANOVA with repeated measures. For a medium effect size (partial eta squared = 0.06) [35], assuming a statistical power of 80% and a two-sided significance level (α) of 0.05, a total of 34 subjects were required. Considering an attrition rate of 30%, the necessary sample size was determined to be 23 per group, resulting in a total of 46 participants.

### 2.6. Data Analysis

The data were analyzed using SPSS version 27.0 (IBM SPSS Statistics, Armonk, NY, USA). A significance level of 0.05 was set for all analyses. The normality of the distribution of continuous data was assessed using the Shapiro–Wilk test. In cases where the data were found to not follow a normal distribution, non-parametric statistics were used to determine statistical significance. Chi-square tests were used for categorical variables, while Mann–Whitney tests were conducted for age and body mass index to evaluate the significance of intergroup differences at baseline.

If all four data sets (2 × 2: data from moxibustion and acupressure groups at baseline and week 8) exhibited a normal distribution, a two-way ANOVA with repeated measures was conducted to assess the significance of intergroup differences in changes from baseline (group-by-time interaction). The factors included in the main effect were time (baseline vs. week 8) and group (moxibustion vs. acupressure). Two-way ANOVA with repeated measures was conducted on the PSQI total score, the three domains of WHOQOL-BREF (physical health, psychological health, and environment), and the hematocrit. Results of some variables, including PSQI, domains of WHOQOL-BREF, and the blood cell count, that were analyzed using non-parametric statistical analyses are presented separately in the tables.

We conducted non-parametric statistical analyses on systolic and diastolic blood pressures, epinephrine, norepinephrine, PSS, the PSQI subscales, the WHOQOL-BREF social relationship domain, and four variables of blood cell counts. In cases where the data did not exhibit a normal distribution, the significance of intragroup differences was tested using paired *t*-tests or Wilcoxon signed-rank tests. To evaluate the significance of intergroup differences, independent *t*-tests or Mann–Whitney tests were performed.

The Pearson correlation coefficient (r) was used to assess the degree of linear association between baseline blood pressure and the magnitude of blood pressure change (week 8–baseline). Additionally, effect size estimates of the statistics were calculated using the following formulas: Partial eta squared (η^2^) for two-way ANOVA with repeated measures; Cohen’s d for *t*-tests; and the r-values for the Wilcoxon signed-rank test and Mann–Whitney test [35,36]. These calculations were performed using SPSS. The effect sizes were interpreted as small (η^2^ = 0.01; d = 0.2), medium (η^2^ = 0.06; d = 0.5), and large (η^2^ ≥ 0.14; d ≥ 0.8) effects for partial eta squared and Cohen’s d [35,37]. For the r-value, it was calculated as the Z statistic divided by the square root of the total sample size or the total number of pairs [36] and interpreted as small (r = 0.1), medium (r = 0.3), and large (r ≥ 0.5) effects [37].

Both per-protocol and intention-to-treat analyses were conducted. In the per-protocol analysis, data were analyzed only for subjects who completed this study (Figure 1). For the intention-to-treat analysis, all randomized subjects were included, and data from all subjects were utilized for the analysis of all outcomes. In cases where values were missing, imputation was performed using regression imputation. Results based on per-protocol analyses are presented in the tables. The results from the intention-to-treat analyses were described at the end of each section of the Results.

## 3. Results

### 3.1. Participant Characteristics

Out of the 46 subjects initially randomized, 21 were in the moxibustion group, and 20 completed this study and were included in the per-protocol analysis. In terms of completing the entire intervention session (40 sessions), 17 subjects in the moxibustion group and 18 subjects in the acupressure group accomplished this requirement. The average completion rate of the sessions was 97.5% in the moxibustion group and 97.3% in the acupressure group.

The subject characteristics are summarized in Table 1. The majority of the subjects were male (65.9%). The mean age of the subjects was 61.7 years with a standard deviation of 10.8 years, and the mean body mass index was 26.0 kg/m^2^ with a standard deviation of 3.7 kg/m^2^. There were no significant intergroup differences in gender proportion or age. However, the acupressure group had a significantly higher body mass index compared to the moxibustion group (*p* < 0.05). Most subjects (82.9%) had a college education or higher. Approximately 95% of the subjects were married, and the majority (95%) were non-smokers. Furthermore, 46.3% of subjects reported being non-drinkers, and 85.4% did not engage in moderate-to-vigorous-intensity exercise. There were no significant intergroup differences in terms of education, marital status, exercise, smoking, or drinking.

Among the medications taken by the subjects, the majority were for managing conditions such as hypertension, diabetes mellitus, and hyperlipidemia. In the moxibustion group, two subjects had prehypertension. Among the 39 hypertensive subjects, 24 individuals were taking antihypertensive medication. Approximately 74% of hypertensive subjects in the moxibustion group (*n* = 14) had uncontrolled hypertension, defined as systolic blood pressure ≥ 140 mmHg or diastolic blood pressure ≥ 90 mmHg, with medication (*n* = 7) or without medication (*n* = 7). In the acupressure group, 90% of subjects (*n* = 18) had uncontrolled hypertension, with medication (*n* = 10) or without medication (*n* = 8).

In addition, there were no significant intergroup differences in systolic and diastolic blood pressures, PSS, levels of epinephrine and norepinephrine, and all variables of complete blood count, PSQI, and WHOQOL-BREF at baseline.

**Table 1:** Participant characteristics.

Variables	Category	Total (*n* = 41)	Moxibustion (*n* = 21)	Acupressure (*n* = 20)	*p* ^a^
Age (years)		61.7 ± 10.8	64.3 ± 9.2	59.1 ± 12.0	0.266 ^b^
Body mass index (kg/m^2^)		26.0 ± 3.7	24.9.5 ± 3.4	27.2 ± 3.8	0.047 ^b^
Gender	Male	27 (65.9)	13 (61.9)	14 (70.0)	0.585
	Female	14 (34.1)	8 (38.1)	6 (30.0)	
Education	≤High school	7 (17.1)	4 (19.0)	3 (15.0)	0.886
	College	21 (51.2)	11 (52.4)	10 (50.0)	
	>College	13 (31.7)	6 (28.6)	7 (35.0)	
Marital status	Single	2 (4.9)	0 (0)	2 (10.0)	0.232
	Married	39 (95.1)	21 (100)	18 (90.0)	
Moderate-to-vigorous exercise	No	35 (85.4)	18 (85.7)	17 (85.0)	1.000
	Yes	6 (14.6)	3 (14.3)	3 (15.0)	
Smoking	No	30 (73.2)	16 (76.2)	14 (70.0)	0.896
	Yes	2 (4.9)	1 (4.8)	1 (5.0)	
	Past smoker	9 (22.0)	4 (19.0)	5 (25.0)	
Drinking	No	19 (46.3)	11 (52.4)	8 ((40.0)	0.702
	Once per month	5 (12.2)	3 (14.3)	2 (10.0)	
	2–4 times/month	9 (22.0)	3 (14.3)	6 (30.0)	
	2–3 times/week	5 (12.2)	2 (9.5)	3 (15.0)	
	≥4 times/week	3 (7.3)	2 (9.5)	1 (5.0)	
Hypertension medication	No (hypertensive)	17 (41.5)	7 (33.3)	8 (40.0)	0.360
	No (pre-hypertensive)	2 (4.9)	2 (9.5)	0 (0.0)	
	Yes	24 (58.5)	12 (57.1)	12 (60.0)	
Other medication	Hyperlipidemia	8 (19.5)	2 (9.5)	6 (30.0)	Multiple responses
	Diabetes mellitus	8 (19.5)	5 (23.8)	3 (15.0)	
	Others	4 (9.8)	2 (9.5)	2 (10.0)	

Data are presented as mean ± standard deviation or *n* (%). ^a^ The significance of the difference between the groups was tested using the Chi-square test. ^b^ The significance of the difference between the groups was tested using the Mann–Whitney U test.

### 3.2. Changes in Blood Pressure and Stress

As shown in Table 2, based on the per-protocol analyses using the Wilcoxon signed-rank test and a paired *t*-test, systolic blood pressure significantly decreased in the moxibustion group (*p* < 0.001) and the acupressure group (*p* < 0.05) after the 8-week intervention. Similarly, diastolic blood pressure significantly decreased in both the moxibustion group (*p* < 0.01) and the acupressure group (*p* < 0.01). Results from the Mann–Whitney U test and independent *t*-test indicated that there were no significant intergroup differences in the extent of changes from baseline in systolic and diastolic blood pressures (Table 2).

Correlation analysis of pooled data from two groups (*n* = 41) revealed that the magnitude of reduction in blood pressure was significantly correlated with baseline blood pressure (r = −0.64; *p* < 0.001) and diastolic blood pressure (r = −0.70; *p* < 0.001), indicating that subjects with higher baseline blood pressures experienced a greater blood pressure reduction with the treatments.

After 8 weeks of intervention, no significant changes were observed within either group for epinephrine or norepinephrine levels. However, a significant decrease in perceived stress, as measured by the PSS, was observed only in the moxibustion group (*p* < 0.05). Nonetheless, there were no significant intergroup differences in the extent of change from baseline for PSS (Table 2).

In the intention-to-treat analyses for the outcomes of blood pressure and stress, there were small changes in the *p*-values and mean values of the variables compared to those in the per-protocol analysis. However, the findings remained consistent and did not differ from those of the per-protocol analysis.

### 3.3. Changes in Sleep Quality and Quality of Life

The results from the per-protocol analyses regarding intragroup and intergroup differences in PSQI subscales are presented in Table 3. According to the Wilcoxon signed-rank tests, no significant intragroup changes were observed in sleep duration, habitual sleep efficiency, sleep disturbance, use of sleeping medication, or daytime dysfunction in both groups. Furthermore, the Mann–Whitney U tests indicated that there were no significant intergroup differences in any of the PSQI subscales.

Table 4 presents the results from a two-way ANOVA with repeated measures, examining the main effects of time and group as well as the time-by-group interaction effect. The significance of the change in the PSQI total score and three domains of the WHOQOL-BREF was tested using this two-way ANOVA with repeated measures. As indicated in Table 4, the main effects of time and group, as well as the group-by-time interaction, were not significant for the PSQI total score. However, there were significant improvements in the physical health domain (*p* < 0.01) and environment domain (*p* < 0.05) of the WHOQOL-BREF in both groups, indicating a significant main effect of time without the presence of an interaction effect.

As presented in Table 3, the result from the Wilcoxon signed-rank test demonstrated a significant improvement in the social relationship domain (*p* < 0.05) in the moxibustion group. However, no significant improvement was observed in the acupressure group. Nevertheless, the intergroup difference in the change from baseline did not reach statistical significance based on the Mann–Whitney U test.

In the intention-to-treat analysis for all PSQI and WHOQOL-BREF variables, there were slight changes in the *p*-values and mean values compared to those in the per-protocol analysis. However, the findings from the intention-to-treat analysis remained consistent with those of the per-protocol analysis.

### 3.4. Adverse Events

Reported adverse events included blister occurrences (*n* = 1) in the moxibustion group and skin redness (*n* = 1) in the acupressure group. In both the per-protocol analysis (Table 4 and Table 5) and the intention-to-treat analysis, the variables of the blood cell count did not show significant changes in either group.

**Table 2:** Intragroup and intergroup differences in blood pressure, stress hormones, and perceived stress.

Variables	Group	Baseline		Week 8		*p* ^a^	Effect Size ^c^	Week 8–Baseline		*p* ^e^	Effect Size ^c^
		Mean ± SD	Median (IQR)	Mean ± SD	Median (IQR)			Mean ± SD	Median (IQR)		
Systolic blood pressure (mmHg)	Moxibustion	144.3 ± 21.3	135.0 (130.0–150.0)	130.5 ± 13.2	130.0 (120.0–140.0)	0.000 ***	0.78	−13.8 ± 12.5	−10.0 (−20.0–−2.5)	0.289	0.17
	Acupressure	140.5 ± 13.2	135.0 (130.0–150.0)	133.0 ± 14.9	130.0 (120.0–140.0)	0.024 *	0.50	−7.5 ± 16.2	−10.0 (−20.0–0.0)		
Diastolic blood pressure (mmHg)	Moxibustion	94.1 ± 15.8	90.0 (80.0–105.0)	81.9 ± 10.8	80.0 (75.0–90.0)	0.001 **	0.76	−12.1 ± 11.2	−10.0 (−20.0–−5.0)	0.391 ^f^	0.27 ^d^
	Acupressure	91.8 ± 8.2	90.0 (90.0–93.8)	82.5 ± 7.2	80.0 (80.0–90.0)	0.002 **	0.69	−9.3 ± 10.0	−17.5 (−20.0–0.0)		
Epinephrine (pg/mL)	Moxibustion	56.0 ± 23.4	57.5 (36.1–71.5)	44.0 ± 17.6	41.9 (29.9–50.3)	0.085 ^b^	0.40 ^d^	−12.0 ± 30.4	−14.6 (−34.2–4.9)	0.823 ^f^	0.07 ^d^
	Acupressure	49.9 ± 21.2	53.3 (29.1–70.6)	39.7 ± 17.4	35.6 (28.6–45.0)	0.076	0.40	−10.2 ± 22.1	−8.0 (−29.0–7.9)		
Norepinephrine (pg/mL)	Moxibustion	587.9 ± 285.2	483.0 (420.0–605.2)	505.1 ± 224.7	458.1 (360.9–573.6)	0.122	0.34	−82.8 ± 289.6	−98.3 (−174.0–87.1)	0.639	0.07
	Acupressure	557.8 ± 294.8	503.9 (338.6–638.1)	482.3 ± 188.1	486.3 (369.4–589.6)	0.370	0.20	−75.5 ± 231.5	−17.1 (−192.4–125.6)		
Perceived stress	Moxibustion	15.7 ± 4.1	15.0 (13.0–19.0)	12.8 ± 4.7	12.0 (8.5–15.5)	0.028 *	0.48	−2.9 ± 5.2	−3.0 (−5.0–1.5)	0.355 ^f^	0.29 ^d^
	Acupressure	14.2 ± 3.7	14.0 (12.0–16.0)	12.7 ± 5.3	12.5 (8.0–16.5)	0.185 ^b^	0.31 ^d^	−1.5 ± 4.7	−0.5 (−5.8–1.8)		

Data used for per-protocol analyses are presented. The Moxibustion and Acupressure groups consist of 21 and 20 subjects, respectively. SD: Standard deviation; IQR: Interquartile range (25th–75th percentile). ^a^ The significance of the difference between baseline and week 8 in each group was tested by the Wilcoxon signed-rank test. ^b^ The significance of the difference between baseline and week 8 in each group was tested by a paired *t*-test. ^c^ The Cohen’s d effect size. ^d^ The r effect size. ^e^ The significance of the difference was tested by the Mann–Whitney U test. ^f^ The significance of the difference between the groups was tested using an independent *t*-test. * *p* <0.05, ** *p* < 0.01, *** *p* < 0.001.

**Table 3:** Intragroup and intergroup differences in the PSQI subscales and the WHOQOL-BREF social relationship domain.

Variables	Group	Baseline		Week 8		*p* ^a^	Effect Size ^d^	Week 8–Baseline		*p* ^e^	Effect Size ^d^
		Mean ± SD	Median (IQR)	Mean ± SD	Median (IQR)			Mean ± SD	Median (IQR)		
PSQI Subscales											
Subjective sleep quality	Moxibustion	1.1 ± 0.7	1.0 (1.0–2.0)	0.8 ± 0.5	1.0 (0.5–1.0)	0.071	0.39	−0.3 ± 0.8	0.0 (−1.0–0.0)	0.418	0.13
	Acupressure	1.2 ± 0.8	1.0 (1.0–2.0)	1.1 ± 0.6	1.0 (1.0–1.0)	0.564	0.13	−0.1 ± 0.8	0.0 (−1.0–0.0)		
Sleep latency	Moxibustion	1.0 ± 0.9	1.0 (0.0–2.0)	0.8 ± 0.6	1.0 (0.0–1.0)	0.109	0.35	−0.3 ± 0.8	0.0 (−1.0–0.0)	0.499	0.11
	Acupressure	1.0 ± 0.7	1.0 (0.3–1.8)	0.9 ± 0.7	1.0 (0.0–1.0)	0.180	0.30	−0.2 ± 0.5	0.0 (0.0–0.0)		
Sleep duration	Moxibustion	1.1 ± 0.7	1.0 (1.0–1.5)	1.1 ± 0.5	1.0 (1.0–1.0)	0.739	0.07	−0.0 ± 0.7	0.0 (−0.5–0.0)	0.383	0.14
	Acupressure	0.9 ± 1.1	1.0 (0.0–1.0)	1.0 ± 0.9	1.0 (0.3–1.0)	0.564	0.13	0.1 ± 0.8	0.0 (0.0–1.0)		
Habitual sleep efficiency	Moxibustion	0.4 ± 0.7	0.0 (0.0–1.0)	0.2 ± 0.5	0.0 (0.0–0.0)	0.470	0.16	−0.1 ± 0.9	0.0 (−0.5–0.0)	0.621	0.08
	Acupressure	0.3 ± 0.7	0.0 (0.0–0.0)	0.3 ± 0.6	0.0 (0.0–0.0)	0.739	0.07	−0.1 ± 0.7	0.0 (0.0–0.0)		
Sleep disturbance	Moxibustion	1.2 ± 0.6	1.0 (1.0–2.0)	1.3 ± 0.6	1.0 (1.0–1.5)	0.527	0.14	0.1 ± 0.7	0.0 (0.0–1.0)	0.232	0.19
	Acupressure	1.1 ± 0.4	1.0 (1.0–1.0)	0.9 ± 0.7	1.0 (0.0–1.0)	0.257	0.25	−0.2 ± 0.6	0.0 (−0.8–0.0)		
Use of sleeping medication	Moxibustion	0.0 ± 0.0	0.0 (0.0–0.0)	0.1 ± 0.2	0.0 (0.0–0.0)	0.317	0.22	0.0 ± 0.2	0.0 (0.0–0.0)	0.972	0.01
	Acupressure	0.0 ± 0.0	0.0 (0.0–0.0)	0.1 ± 0.2	0.0 (0.0–0.0)	0.317	0.22	0.1 ± 0.2	0.0 (0.0–0.0)		
Daytime dysfunction	Moxibustion	1.1 ± 1.0	1.0 (0.0–2.0)	1.0 ± 0.7	1.0 (0.5–1.0)	0.458	0.16	−0.2 ± 1.1	0.0 (−1.0–0.0)	0.872	0.03
	Acupressure	1.2 ± 0.8	1.0 (0.3–2.0)	1.0 ± 0.9	1.0 (0.0–2.0)	0.435	0.17	−0.2 ± 1.0	0.0 (0.0–0.0)		
WHOQOL-BREF domain											
Social relationships	Moxibustion	68.6 ± 10.1	66.7 (60.0–73.3)	72.1 ± 10.2	73.3 (66.7–80.0)	0.044 *	0.44	3.5 ± 7.8	0.0 (−3.3–13.3)	0.915	0.02
	Acupressure	66.3 ± 10.5	66.7 (60.0–73.3)	69.3 ± 12.9	70.0 (60.0–80.0)	0.317 ^b^	0.23 ^c^	3.0 ± 13.1	0.0 (−5.0–16.7)		

Data used for per-protocol analyses are presented. The Moxibustion and Acupressure groups consist of 21 and 20 subjects, respectively. PSQI: Pittsburgh sleep quality index; WHOQOL-BREF: World Health Organization quality of life-brief version; SD: Standard deviation; IQR: Interquartile range (25th–75th percentile). ^a^ The significance of the difference between baseline and week 8 in each group was tested by the Wilcoxon signed-rank test. ^b^ The significance of the difference between baseline and week 8 in each group was tested by a paired *t*-test. ^c^ The Cohen’s d effect size. ^d^ The r effect size. ^e^ The significance of the difference was tested by the Mann–Whitney U test. * *p* < 0.05.

**Table 4:** Results from a two-way ANOVA with repeated measures for PSQI total score, three WHOQOL-BREF domains, and hematocrit.

Variables	Group	Baseline	Week 8	*p* (η^2^)		
				Time	Group	Interaction
PSQI						
Total score	Moxibustion	6.0 ± 2.6	5.2 ± 1.9	0.069	0.684	0.564
	Acupressure	5.6 ± 3.0	5.1 ± 2.5	(0.082)	(0.004)	(0.009)
WHOQOL-BREF domain						
Physical health	Moxibustion	67.9 ± 13.4	72.8 ± 12.2	0.002 **	0.263	0.556
	Acupressure	72.7 ± 12.1	76.1 ± 10.7	(0.225)	(0.032)	(0.009)
Psychological health	Moxibustion	71.1 ± 11.9	73.2 ± 11.8	0.076	0.699	0.703
	Acupressure	69.3 ± 9.3	72.5 ± 10.9	(0.079)	(0.004)	(0.079)
Environment	Moxibustion	68.1 ± 13.9	71.5 ± 13.5	0.035 *	0.669	0.563
	Acupressure	70.4 ± 11.1	72.4 ± 9.9	(0.110)	(0.005)	(0.009)
Complete blood count						
Hematocrit	Moxibustion	40.8 ± 4.7	40.8 ± 4.7	0.961	0.238	0.909
	Acupressure	42.3 ± 3.5	42.3 ± 3.4	(0.000)	(0.035)	(0.000)

Data used for per-protocol analyses are presented. The Moxibustion and Acupressure groups consist of 21 and 20 subjects, respectively. PSQI: Pittsburgh sleep quality index; WHOQOL-BREF: World Health Organization quality of life-brief version; η^2^: Partial eta squared (effect size). * *p* <0.05, ** *p* < 0.01.

**Table 5:** Intragroup and intergroup differences in complete blood count.

Variables	Group	Baseline		Week 8		*p* ^a^	Effect Size ^d^	Week 8–Baseline		*p* ^e^	Effect Size ^d^
		Mean ± SD	Median (IQR)	Mean ± SD	Median (IQR)			Mean ± SD	Median (IQR)		
White blood cells (10^3^/µL)	Moxibustion	6.4 ± 2.0	5.9 (5.4–7.1)	6.3 ± 1.5	5.9 (5.2–7.1)	0.651	0.10	−0.1 ± 1.5	−0.4 (−0.8–0.8)	0.481	0.11
	Acupressure	6.0 ± 1.5	5.9 (5.0–7.2)	6.2 ± 2.2	5.7 (4.9–6.6)	0.422	0.18	0.2 ± 1.7	0.2 (−0.4–0.6)		
Platelets (10^3^/µL)	Moxibustion	248.0 ± 54.7	246.0 (203.0–283.5)	234.7 ± 61.1	248.0 (180.5–284.0)	0.213 ^b^	0.28 ^c^	−13.2 ± 47.2	−9.0 (−27.0–15.5)	0.611	0.08
	Acupressure	241.1 ± 46.8	231.5 (217.5–269.8)	226.9 ± 50.4	234.5 (213.8–254.0)	0.079	0.39	−14.2 ± 30.6	−15.0 (−34.5–11.8)		
Red blood cells (10^6^/µL)	Moxibustion	4.5 ± 0.5	4.6 (4.1–4.8)	4.5 ± 0.5	4.6 (4.2–4.9)	0.737	0.07	0.0 ± 0.2	0.0 (−0.2–0.2)	0.734	0.05
	Acupressure	4.6 ± 0.4	4.6 (4.4–4.9)	4.6 ± 0.4	4.6 (4.3–5.0)	0.484 ^b^	0.16 ^c^	−0.0 ± 0.3	0.0 (−0.2–0.1)		
Hemoglobin (g/dL)	Moxibustion	15.2 ± 6.9	14.0 (12.8–15.3)	13.5 ± 1.7	13.7 (12.8–14.8)	0.423	0.17	−1.7 ± 7.4	−0.2 (−0.5–0.5)	0.296	0.16
	Acupressure	14.1 ± 1.4	14.4 (13.4–15.0)	14.1 ± 1.2	14.1 (13.4–15.2)	1.00 ^b^	0.00 ^c^	0.0 ± 0.9	0.2 (−0.3–0.4)		

Data used for per-protocol analyses are presented. The Moxibustion and Acupressure groups consist of 21 and 20 subjects, respectively. SD: Standard deviation; IQR: Interquartile range (25th–75th percentile). ^a^ The significance of the difference between baseline and week 8 in each group was tested by the Wilcoxon signed-rank test. ^b^ The significance of the difference between baseline and week 8 in each group was tested by a paired *t*-test. ^c^ The Cohen’s d effect size. ^d^ The r effect size. ^e^ The significance of the difference was tested by the Mann–Whitney U test.

## 4. Discussion

This study aimed to evaluate the effects of 8-week moxibustion self-care on blood pressure, stress, sleep quality, and quality of life in hypertensive adults, comparing its effects with those of acupressure self-care. The main finding of this study was that both moxibustion and acupressure self-care were effective in reducing blood pressure and improving quality of life, specifically in relation to physical health and the environment. Interestingly, perceived stress levels and quality of life related to social relationships improved only with moxibustion self-care. However, stress hormones, including epinephrine and norepinephrine, as well as sleep quality, did not show significant changes after the intervention.

Moxibustion has been commonly used as a self-care practice at home in Asian countries for various health conditions [12,38]. However, there are concerns regarding the safety and compliance of self-administered moxibustion due to potential adverse events such as burns and blisters [39]. Interestingly, these skin wounds are generally considered part of the treatment process and are well tolerated by individuals practicing moxibustion [39,40]. Nevertheless, the feasibility of moxibustion therapy as a self-care practice has received limited investigation. Cheung et al. reported trends of improvement in pain and function in older adults with knee osteoarthritis using self-administered electro-moxibustion [41]. While there is a protocol for the self-administration of moxibustion in primary hypertension, further research is warranted [13].

Acupressure is a non-invasive treatment option that can be easily administered by patients themselves via patches and bands [15]. Previous studies, including a systematic review, have reported self-acupressure as an effective self-management method for various health conditions, including stress, sleep disturbance, and quality of life, which were measured in this study [42,43,44,45]. Moxibustion and acupressure share the same therapeutic mechanisms based on the meridian theory [9,13,14]. Therefore, we compared the effects of self-moxibustion with acupressure as a positive control to investigate the feasibility of self-management for hypertension.

Our results demonstrated that both self-administered moxibustion and acupressure therapies led to a similar reduction in systolic and diastolic blood pressures. We initially speculated that moxibustion might have a greater effect on blood pressure compared to acupressure due to its additional merits. Moxibustion involves the use of heat and aroma, which are not present in acupressure [17]. These effects of moxibustion have been theorized to induce beneficial changes in the cardiovascular system and promote mental relaxation, thereby lowering blood pressure [13,16,17]. However, contrary to our expectations, moxibustion and acupressure showed similar effects on reducing blood pressure, suggesting that the stimulation of acupoints itself may contribute to the observed blood pressure reduction. Our findings align with previous studies that have reported decreased blood pressure with both moxibustion [12,16] and acupressure [9,10] administered by practitioners to hypertensive patients.

There is an issue that the pre-treatment blood pressure levels have to be taken into account when comparing the efficacy of different antihypertensive treatments [46]. The phenomenon that the response to treatment depends on the pre-treatment value (Wilder’s principle) is well known, but it is still unclear whether this reflects an actual biological phenomenon or merely a statistical artifact [46]. A similar phenomenon has been observed for the therapeutic response of blood pressure: Patients with higher baseline blood pressures experienced a greater reduction in systolic and diastolic blood pressures with antihypertensive drugs, demonstrating that the baseline level significantly influences the magnitude of the antihypertensive effect of the treatments [47,48]. Similarly, our results showed that the higher the baseline blood pressure, the greater the magnitude of reduction in systolic and diastolic blood pressures. This indicates that patients with higher blood pressure experienced a greater fall in blood pressure with the treatments than those with less severe hypertension. In this context, when comparing two therapeutic responses, the disparity in treatment effects could be linked to differences in pre-treatment levels [46]. Therefore, confirming differences in baseline values appears to be necessary when comparing the therapeutic effects of the two methods. In the present study, there were no significant differences in the baseline systolic and diastolic blood pressures.

Many hypertensive patients are not receiving treatment, and a significant number of patients have uncontrolled high blood pressure exceeding 140/90 mmHg [2,6]. Achieving optimal blood pressure levels has additional health benefits, with the lowest risk of cardiovascular disease and mortality observed at a systolic blood pressure of 120 to 124 mmHg [49]. Every 10 mmHg decrease in systolic blood pressure corresponds to a proportional reduction in the risk of cardiovascular disease and mortality [49]. Some patients struggle with uncontrolled blood pressure despite medication and find it challenging to adhere to long-term antihypertensive treatment due to side effects that negatively impact their quality of life [1,12].

In our study, subjects easily learned and applied the techniques of moxibustion and acupressure after a short training course. The dropout rate was low, and overall session adherence at home was excellent. Our results indicate that both therapies can be effective self-care methods for lowering blood pressure with satisfactory adherence. Taking all these aspects into consideration, self-care practices involving moxibustion and acupressure can serve as additional treatment options for blood pressure control in pre-hypertensive and hypertensive patients, complementing conventional treatments.

Previous research has implicated stress as a risk factor for hypertension [3,19,20]. Hypertensive subjects have been found to exhibit increased sympathetic activity compared to normotensive subjects [21]. Based on these findings, we hypothesized that the beneficial effect of moxibustion on blood pressure might be related to changes in stress levels to some extent. In our study, we observed a decrease in perceived mental stress (as measured by the PSS) only in the moxibustion group. However, the magnitude of this change was similar to that observed in the acupressure group.

The effect of moxibustion on stress, including stress hormones and sympathetic activity, has primarily been investigated in stress-induced animal models [23,24]. In terms of clinical studies, there has been only one study examining the effect of moxibustion on stress-related variables [16]. Shin et al. reported that a 4-week moxibustion therapy did not lead to changes in sympathetic activity, as measured by heart rate variability, in prehypertensive and hypertensive patients [16]. The knowledge regarding the effect of moxibustion on mental stress is very limited. Therefore, further studies are necessary to gain a clearer understanding of its clinical effectiveness on stress. While additional research is needed to support our results, our findings provide an initial insight into the involvement of mental stress and stress hormones in the effect of moxibustion on hypertension.

The effect of moxibustion on sleep in hypertensive patients is not well understood. In our study, we observed that the total and subscale scores of the PSQI did not significantly change in both groups after the intervention. Similarly, Shin et al. also reported no effects of a 4-week moxibustion therapy on the total score of the PSQI in prehypertensive and hypertensive patients, although they did not provide the results for the PSQI subscales [16].

The results of our study demonstrated that both the moxibustion and acupressure groups experienced improvements in the domains of physical health and environment, whereas social relationships showed improvement only in the moxibustion group. In contrast, Shin et al. [16] did not observe any effects of a 4-week moxibustion therapy on quality of life in prehypertensive and hypertensive patients. This discrepancy may be attributed to the shorter duration of their treatment session (4 weeks) and the utilization of a different measurement method for assessing quality of life. It is possible that a longer treatment session may be necessary to observe improvements in quality of life with moxibustion in hypertensive patients.

This study has several limitations. It should be acknowledged that some subjects may not have strictly adhered to the protocol guidelines for moxibustion and acupressure due to the nature of self-administration. This potential non-compliance could have influenced the study’s results. Thus, additional research is warranted to compare the effects of self-administered moxibustion and acupressure with those performed by practitioners. Nonetheless, this study represents the first investigation assessing the feasibility of moxibustion and acupressure as self-management methods for hypertension. Additionally, our findings provide the first evidence suggesting that moxibustion therapy may have potential clinical benefits for reducing stress.

## 5. Conclusions

This study demonstrated that self-administered moxibustion and acupressure therapies resulted in a significant decrease in systolic and diastolic blood pressures among hypertensive patients, with no significant difference between the two. The reported adverse events were minor, and the participants showed good adherence to the therapies. These findings indicate that both moxibustion and acupressure can be effective self-management methods for reducing blood pressure in hypertensive patients. Additionally, improvements in quality of life, specifically related to physical health and the environment, were observed in both therapy groups. It is noteworthy that only moxibustion showed improvements in perceived stress levels and quality of life related to social relationships; however, further studies are needed to support these results. Overall, these findings suggest that both moxibustion and acupressure therapies hold promise as self-care methods for the prevention and management of hypertension and its associated symptoms.

## Data Availability

The data presented in this study are available on request from the corresponding author.
